# Improving Predictions of Multiple Binary Models in ILP

**DOI:** 10.1155/2014/739062

**Published:** 2014-02-13

**Authors:** Tarek Abudawood

**Affiliations:** Machine Learning & Medical Informatics Lab, Computer Research Institute, King Abdulaziz City for Science and Technology (KACST), Riyadh 11442, Saudi Arabia

## Abstract

Despite the success of ILP systems in learning first-order rules from small number of examples and complexly structured data in various domains, they struggle in dealing with multiclass problems. In most cases they boil down a multiclass problem into multiple black-box binary problems following the one-versus-one or one-versus-rest binarisation techniques and learn a theory for each one. When evaluating the learned theories of multiple class problems in one-versus-rest paradigm particularly, there is a bias caused by the default rule toward the negative classes leading to an unrealistic high performance beside the lack of prediction integrity between the theories. Here we discuss the problem of using one-versus-rest binarisation technique when it comes to evaluating multiclass data and propose several methods to remedy this problem. We also illustrate the methods and highlight their link to binary tree and Formal Concept Analysis (FCA). Our methods allow learning of a simple, consistent, and reliable multiclass theory by combining the rules of the multiple one-versus-rest theories into one rule list or rule set theory. Empirical evaluation over a number of data sets shows that our proposed methods produce coherent and accurate rule models from the rules learned by the ILP system of Aleph.

## 1. Introduction

Inductive logic programming is a branch of machine learning that is concerned with learning logic programs inductively from examples in structural domains [1, 2]. ILP algorithms, such as FOIL [3], PROGOL [4], and Aleph [5], induce theories from a small number of examples to be generalised over the entire population of examples through learning first-order clauses (or rules) that mostly take the form of Horn clauses. A Horn clause is a disjunction of literals (atomic formulae) with at most one positive literal. Most ILP algorithms use a programming language called  PROLOG  (PROLOG was originally created by Alain Colmerauer and Robert Kowalski in 1972) which stands for PROgramming in LOGic. In PROLOG, theories (or programs) are expressed using a collection of Horn clauses [6]. In fact a theory, whether it is an input or output, is understood and interpreted as a conjunction of Horn clauses. We often refer to a Horn clause as a rule. Unlike the propositional logic framework, first-order logic (FOL) framework allows the use of variables and structural literals in addition to the use of functional literals.

While first-order decision tree learners, such as TILDE [7], can learn from examples of multiple classes, first-order rule learners in ILP typically learn rules from two classes only (positive and negative examples as we pointed out earlier). Despite their ability to learn from complex structured data and build effective classification models in a range of domains, they struggle, unfortunately, in dealing with multiclass problems. In most situations they reduce a multiclass problem into multiple binary problems following the pairwise one-versus-one or one-versus-rest binarisation techniques.

Aleph, as a case in point, can learn a multiclass theory in the one-versus-rest paradigm where the outcome of its induction can be seen as a combination of several black-box models. Each model induces rules for one specific (positive) class, and a default rule is added to predict the remaining classes.

As discussed earlier, many ILP rule learning systems including Aleph, PROGOL, and FOIL can only induce binary theories and multiclass theories are obtained by converting a multiclass problem into several binary problems. The rules of the final model are, in practice, a combination of independent multiple binary theories. Inductive Logic Constraint (ICL) [8] upgraded the propositional CN2 [9] to handle multiclass first-order theories. While most of the ILP systems implement the covering (separate-and-conquer) approach, TILDE implements a divide-and-conquer approach and induces a single first-order logic multiclass theory that takes a form of decision tree. Tree models handle multiple classes naturally.

Several papers suggested different approaches of dealing with multiple binary models [10–16]. A comparison of many such approaches was made in [15] not only suggesting a superiority of the one-versus-rest approach in general but also pointing out that the choice of the binarisation technique makes little difference once we learn good binary models.

Nevertheless, we challenge the suitability of the one-versus-rest approach for first-order rule learners. This is because there is a strong bias towards the negative classes leading to unrealistic estimates of predictive power. Moreover, the lack of integrity between the different binary models results in inconsistent predictions.

At the end of the introductory section we would like to outline the remaining sections of the article.  Section 2 investigates the reliability and consistency of one-versus-rest binary models and illustrates the difference with a proper multiclass model. The reliability reflects how much one can rely on the quality of a one-versus-rest binary model while the consistency reflects how consistent are the predictions of multiple one-versus-rest binary models. In Section 3 we investigate several methods to overcome the problems of the current application of one-versus-rest technique in ILP rule learners. Additionally, we study and illustrate a simple method of representing the rules in a concept lattice in Section 4. We experimentally demonstrate the performance of our suggested methods in Section 5 and compare them to the standard binary method of Aleph. In the final section we summarise the work and discussion presented in this journal paper and draw the conclusion.

## 2. Multiclass versus Multimodel Predictions

In machine learning accuracy is widely used for comparing and assessing the classification performance. Hence many researchers report their results in terms of accuracy and compare their results with accuracies of other algorithms. The accuracy of a model can be interpreted as the expectation of correctly classifying a randomly selected example.

**Figure 1:**
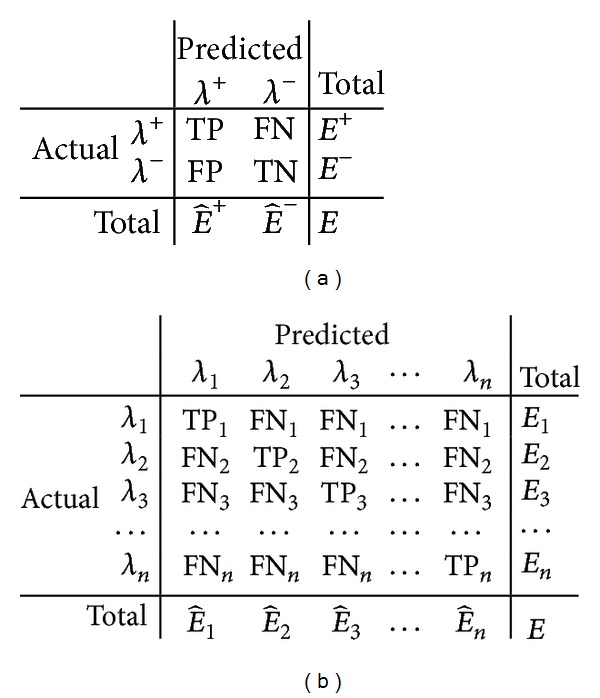
Contingency tables for a binary model (a) and a multiclass model (b), where *λ*, *E*, and $\hat{E}$ reflect a class label, actual number of examples, and predicted number of examples of the class indicated to by the subscript or the superscript, respectively. The TP and FN are the numbers of examples correctly and incorrectly predicted with respect to the class of interest (i.e., *λ* ^+^). TN and FP are the numbers of examples correctly and incorrectly predicted with respect to the negative class in a binary problem only. *n* reflects the number of classes in a multiclass problem.

With respect to the notation explained in Figure 1, let us introduce the following definitions.

**Definition 1 (recall)**

The recall of a given class  *λ*_*i*_, denoted as  Recall_*i*_  or  Recall_*i*_^+^, is the proportion of examples of class  *λ*_*i*_  that is correctly classified by a model (Recall_*i*_ = TP_*i*_/*E*_*i*_). The negative recall of class  *λ*_*i*_, denoted as  Recall_*i*_^−^, is the proportion of examples of class  *λ*_*i*_  incorrectly classified (Recall_*i*_^−^ = 1 − TP_*i*_/*E*_*i*_). In case of two classes, positive and negative, we denote the recall of the positive class as  Recall^+^ = TP/*E* ^+^  and of the negative class as  Recall^−^ = TN/*E* ^−^.

**Definition 2 (accuracy)**

Given two classes,  *λ* ^+^  and  *λ* ^−^, the binary accuracy of a model is defined as

(1) Accuracybin=TP+TNE=E+E·TPE++E−E·TNE−=E+ERecall++E−ERecall−;

that is, binary accuracy is a weighted average of the positive and negative recall, weighted by the class prior. This extends to multiple classes:

(2) Accuracy=∑i=1nTPiE=∑i=1nEiETPiEi=∑i=1nEiERecalli+.

For this reason we sometimes refer to accuracy as (weighted) average positive recall.

**Definition 3 (multimodel accuracy)**

Given  *n*  classes and  *n*  one-versus-rest models, one for each class, the multimodel accuracy is defined as the average binary accuracy of the  *n*  models:

(3) Accuracymm=1n∑i=1n(Ei+ERecalli++Ei−ERecalli−).

The following result is worth noting.

**Lemma 4**

The accuracy of a single multiclass model is not equivalent to the multimodel accuracy of the one-versus-rest models derived from the multiclass model.

**Proof**

One has

(4) Accuracymm=1n∑i=1n(Ei+ERecalli++Ei−ERecalli−)

(5) =1n∑i=1nEi+ERecalli++1n∑i=1nEi−ERecalli−

(6) =1nAccuracy+1n∑i=1nEi−ERecalli−.

In going from (5) to (6) in the above equations, we rely on the fact that the one-versus-rest models are derived from a single multiclass model. If the case is different (unlike the case in Aleph, for instance), then weighted average positive recall is not the same as accuracy, which compounds the issue.

It can be seen from Lemma 4 that the two accuracies are not the same. Accuracy of a multiclass model relies on the positive recalls weighted by the class priors, while the average accuracy of multiple binary models relies on the recalls of both classes where the importance of the positive recalls is decreased  *n*  times. Hence, there is an increase of the importance of classifying a negative example  *n*  times. It is clear that the average accuracy of the binary models is 1.5 times more than the accuracy of the multiclass model because the weight of the negative class is twice the weight of the positive class. When having a proper multiclass model, there are only credits for classifying examples correctly. Averaging the positive and negative recalls for multiple one-versus-one theories could be misleading but it is even more harmful when it comes to one-versus-rest theories as the problem is propagated.

Another problem arising when inducing multiple independent binary theories is the lack of integrity between the predictions of the different binary theories. This may cause an example to have different possible predictions in several contingency tables because each model produces predictions independently of the others. The predictions of the models on each example should be consistent. For instance, by considering  *n*  one-versus-rest models where each model is trained to predict one class as positive, then the prediction for an example  *x*  on the  *i*th model should be consistent with its prediction on the  *j*th model;$\;{\hat{\lambda }}_{i}(x)\;$is + ve  and$\;{\hat{\lambda }}_{j}(x)\;$is − ve  for  all  *j* ≠ *i*, where$\;{\hat{\lambda }}_{i}(x)\;$and$\;{\hat{\lambda }}_{j}(x)\;$express the prediction of the  *i*th and the  *j*th binary model, respectively, for example,  *x*.

If the predictions are inconsistent then such conflicts need to be solved to ensure the consistency in the predictions for each example in all models. There are some classification methods that use all one-versus-rest models but resolve these collisions by obtaining  *n*  scores from each one of the  *n*  models and the model with the maximum score wins the prediction [15, 16]. A rule learner such as  CN2  learns ordered rule lists in one of its settings to avoid such conflicts. In pairwise techniques, voting methods [10, 11, 13, 14] can be considered to integrate the predictions.

The discussion about unreliability and inconsistency holds generally when employing one-versus-rest technique in any learning system but we are emphasising the importance of this issue particularly in ILP binary rule learning systems such as Aleph. This is due to the fact that we only induce rules for the positive class in each one-versus-rest model, while a default rule that always predicts the negative class is added in case an example can not be classified by any induced rule. The default rule gets credits for not classifying negative examples which makes it easy to obtain high negative recalls without inducing any rules for the negatives (empty theories) (an empty theory is a theory where a binary rule learner fails to induce any rule for the positive examples) and just predict the negative class being the majority class. Hence, there is a need to integrate the different binary models of such rule learning systems in order to ensure that high reliability and consistency of their model predictions are met.

## 3. Improved Learning of Multiclass Theories

In this section we investigate how one could improve the reliability of the all one-versus-rest theories in ILP by combining their binary models into a single rule list (Multiclass Rule List) or rule set model (Multiclass Rule Set Intersection and Multiclass Rule Set Union). Our approach is different from the other first-order rule learning approaches in various respects. First, it does not treat the  *n*  various models as independent black-box models but instead combines the rules of all the models into a single model. Secondly, there is only one default rule and the class of the default rule is determined probabilistically according to the distribution of the uncovered training examples of all the classes. Finally, a single prediction is obtained for each example in one multiclass contingency table.

### 3.1. Multiclass Rule List Theories

In any rule list model, the rules are ordered in the final theory according to a certain criterion. When an unseen example is encountered, the rules are tried one by one in the order of the list and the first rule that fires determines the class of the example. So the key idea is to have a sensible criterion to determine the order of the rules in the list. This can be achieved simply by evaluating rules and assigning their numerical scores that reflect their significance. If we have rules induced by  *n* one-versus-rest models for each one of the  *n*  classes, we need a multiclass scoring function to achieve this goal. Luckily several multiclass evaluation measures have been proposed earlier in [17]. They can be used to evaluate all rules over the multiple classes. We then can prioritise the rules which have been obtained from  *n*  one-versus-rest models based on their multiclass scores to build a Multiclass Rule List (MRL) model. This is similar to prioritising the subgroup rules before building a subgroup tree in [18]. We adopted Chi-Squared  (*x* ^2^(*r*) = ∑_*i*=1_^*n*^[*e*_*i*_ *E* − *eE*_*i*_]^2^/*eE*_*i*_(*E* − *e*))  from the work of [17] in our experiments.

*MRL*. In this method, after learning rules for all classes, the rules are reordered on decreasing *x* ^2^. The ties are broken randomly. If a rule is added to the rule list, then all examples it covers are removed from the training set and the rest of the rules are reevaluated based on the remaining examples until no further rule is left. At the end, a single default rule is assigned predicting the majority class from the distribution of the uncovered examples.

### 3.2. Multiclass Rule Set Theories

In a rule set model, the rules are unordered and the class of a new example is determined based on the training statistics of all rules that fire for that particular example. For instance, the  CN2  propositional rule learner learns a rule set model, in one of its two settings, and tags the rules with their coverage distribution on all the classes. If a new example is to be classified,  CN2  sums up the coverage of all rules that fire over each class and the class with the highest coverage wins. This approach has been adapted by  ICL  first-order rule learner [8]. We propose two methods to handle multiclass rule set theories, the Multiclass Rule Set Intersection (MRSI) method and the Multiclass Rule Set Union (MRSU) method. The descriptions of the two methods are discussed below. Later we will compare our approaches to our upgraded version of Aleph that handles probabilities similarly to  CN2  and  ICL.

*MRSI.* In  MRSI  every rule from the multiple one-versus-rest models is evaluated over the entire training set once, and the identifiers of the examples they cover are stored. A default rule is formed based on the majority class of the uncovered training examples. If a new example is to be classified, all the rules are tried. For those rules that fire, we determine the intersection of their training set coverage using the example identifiers such that the examples in the set are not covered by rules that do not fire. The class distribution of this set gives us the empirical (training) probability on each class. The probability of a test example  *x*_te_  of belonging to class  *λ*_*i*_  with respect to the  MRSI  method can be formalised as follows:

(7) pMRSI(xte ∣ λi)=|⋂u=1,cover(ru,xte)|R|coverage(ru)∩Xi∖⋃u=1,¬cover(ru,xte)|R|coverage(ru)||⋂u=1,cover(ru,xte)|R|coverage(ru)∖⋃u=1,¬cover(ru,xte)|R|coverage(ru)|,

where  cover(*r*_*u*_, *x*_te_)  is a boolean function that is activated if the *u*th rule fires for the testing example  *x*_te_  and  coverage(*r*_*u*_)  is a function that returns the subset of training examples covered by the  *u*th rule. The class with the maximum probability is predicted for the example. Again the ties are broken randomly. In the case of an empty intersection, the majority class is assigned to the example.

*MRSU*. The  MRSU  method differs from the  MRSI  method as it determines the class of a new example based on the union of the training coverage of all rules that cover the new example, instead of the intersection. The probability of a test example *x*_te_ of belonging to class  *λ*_*i*_  with respect to the  MRSU  method can be formalised as follows:

(8) pMRSU(xte ∣ λi) =|⋃u=1,cover(ru,xte)|R|coverage(ru)∩Xi||⋃u=1,cover(ru,xte)|R|coverage(ru)|.

The  MRSU  method is closer in spirit to the  CN2  method, which adds up the coverage of all rules that fire. However, by using example identifiers we avoid doublecounting of examples that are covered by several rules, which means that we obtain proper empirical probabilities rather than  CN2's estimates.

To illustrate those two methods let us consider Example 5. If a new testing example *x*_te_ is found to be covered by the following set of rules  {*r*_2_, *r*_3_}, then the probability distribution over the three classes is  [0/2 = 0.0, 0/2 = 0.0, 2/2 = 1.0], respectively, for  MRSI. As for  MRSU  the probability distribution is  [1/10 = 0.1, 4/10 = 0.4, 5/10 = 0.5], respectively. With regard to  MRL  method, class  *λ*_3_  is predicted, for example, *x*_te_, because the *x* ^2^ score of  *r*_3_  is higher than  *r*_2_. Alternatively we can predict the class probabilistically for  MRL  method based on the coverage distribution of the first rule that fires but this is always going to be the majority class originally predicted by the rule.

**Example 5**

**Figure 2:**
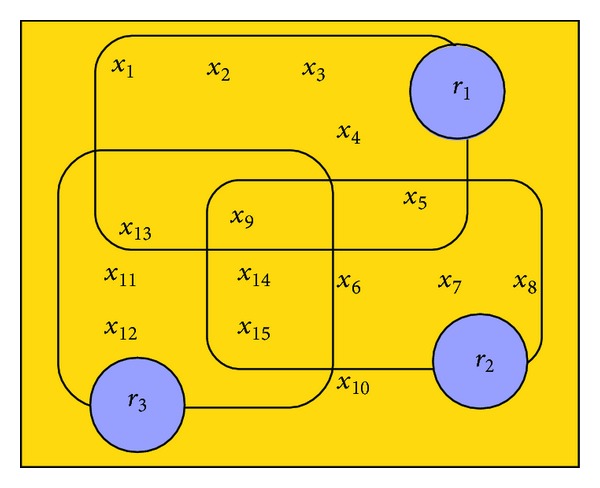
Illustrative figure showing the coverage of three rules and their overlaps over a problem of 15 examples belonging to three classes that corresponds to Table 1.

Simple example illustrating  MRL, MRSI, and  MRSU  methods (this example is borrowed from Abudawood's Ph.D. thesis [19]). Below we give hypothetical three-class problems,  {*λ*_1_, *λ*_2_, *λ*_3_}, of 5 examples each,  {*x*_1_,…, *x*_15_}, and a model of three rules induced on them. The predicted class and the coverage information as well as the *x* ^2^ evaluation scores of the rules are shown in Table 1.  Figure 2 illustrates their coverage and their overlaps.

One has

(9) X1={x1,x2,x3,x4,x5} labelled  with  class  λ1,X2={x6,x7,x8,x9,x10} labelled  with  class  λ2,X3={x11,x12,x13,x14,x15} labelled  with  class  λ3,X=X1∪X2∪X3.

**Figure 3:**
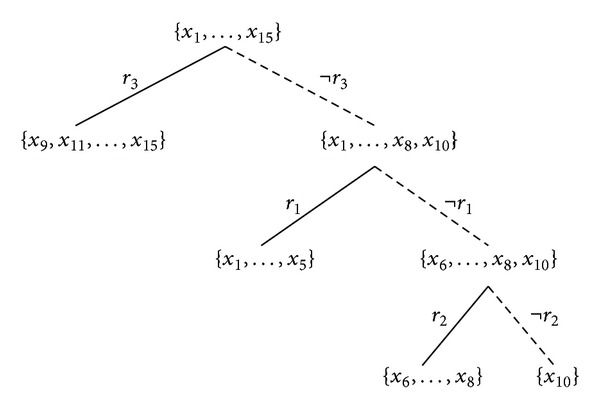
A rule list model that corresponds to  MRL  method of Example 5.

**Figure 4:**
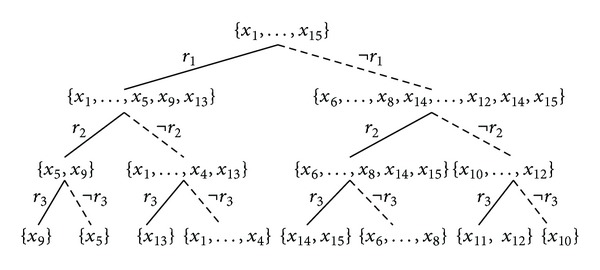
A rule tree model that corresponds to  MRSI  method of Example 5.

**Figure 5:**
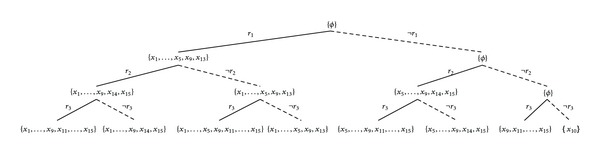
A rule tree model that corresponds to  MRSU  method of Example 5.

In fact all the three methods can be illustrated by drawing a rule list or a rule tree. A rule list corresponds to  MRL  method which is very similar to the conventional decision list (ordered set of rules) model, while a rule tree can be seen as unordered rule set model and hence it is suitable to demonstrate our proposed rule set-based methods,  MRSI  and  MRSU.  Figure 3 illustrates the use of  MRL  method in building a predictive model for Example 5. Figures 4 and 5 show illustrations of the use of  MRSI  and  MRSU  methods, respectively, to create predictive models on the same example.

It deserved to be mentioned that a rule list can be seen as a special type of a rule tree where the node branching is restricted to a left or right branching only in the former one. The construction of a rule tree involves placing a single rule at each single level. In  MRL  and  MRSI  we start building the rule list or rule tree by having all training examples at the root node, and adding a new rule causes the examples at each node to be split into two new nodes reflecting covered and uncovered subset of examples by the new rule on their parent's examples. In  MRSU, however, we start with the empty set of examples at the root and instead of splitting we merge the examples covered by multiple rules such that a leaf will contain all examples covered by a chain set of rules.

## 4. Multiclass Theories and Formal Concept Analysis

In this section we introduce the notion of formal concept lattice in rule learning context and use it to visualise and explore rules, examples, and their binary coverage relationship. We also draw the link between binary trees and formal concept lattice at the end of this section.

### 4.1. Formal Concept Analysis in Rule Learning Context

Formal Concept Analysis (FCA) gains an increasing attention in the field of artificial intelligence and several authors [20–22] have employed it in the machine learning field. It is based on an order theory in mathematics where hypotheses (concepts) and their relationships can be represented in a lattice, called a concept lattice.  FCA  can structure a lattice in a simple way showing how a set of rules are related to each other based on their coverages. It has been used for structuring, exploring, and analysing complex knowledge. A thorough investigation and discussion of  FCA  are beyond the scope of this work and the reader is referred to the survey of [23] for more details on  FCA  and its applications.

FCA  only allows boolean features and we will take advantage of such a powerful technique in the rule learning context by regarding classification rules as binary features. The overall idea is that once we have a set of rules obtained by learning the multiple one-versus-rest models in ILP, we could represent them by a concept lattice with the help of the Formal Concept Analysis technique.

The aim is to explore the rules and their partial relations with respect to their coverage over the examples in a simple and compact graph. Such a graph may not be useful to make predictions in a straightforward manner, but it could give an insight on how the combination of multimodel rules may perform before we even use the multiclass methods discussed above. Let us introduce our basic definitions of  FCA  in rule learning context. We would like to draw the reader attention that the following definitions are adaptations of the classical  FCA  definitions found in the literature where the attributes are simply replaced with rules.

**Definition 6 (formal context)**

Let  *R*  be a set of first-order  rules  (rules can be induced using a first-order or a propositional rule learner),  *X*  a set of examples, and  *G*⊆*X* × *R*  a relation such that  (*x*, *r*) ∈ *G*⇔  *x* ∈ *X*  is covered by  *r* ∈ *R*. A formal context  *K*  is then the triple  (*X*, *R*, *G*).

**Definition 7 (formal concept)**

Let  *A*⊆*X*, *B*⊆*R*, *A*′ = {*r* ∈ *RxGr*  for  all  *x* ∈ *A*}, and  *B*′ = {*x* ∈ *XxGr*  for  all  *r* ∈ *B*}, then a formal concept is defined to be the pair  (*A*, *B*)  satisfying the following four conditions:  *A*⊆*X*, *B*⊆*R*, *A*′ = *B*, and  *B*′ = *A*.  *A*  is called the extent of the formal concept  (*A*, *B*)  and  *B*  is called the intent of the formal concept  (*A*, *B*).

**Definition 8 (concept lattice)**

The concepts are ordered according to  (*A*_1_, *B*_1_)≥(*A*_2_, *B*_2_)⇔*A*_1_⊇*A*_2_, *B*_2_⊇*B*_1_  in order to form the complete concept lattice of the formal context  *K* = (*X*, *R*, *G*). At the bottom of the lattice we can see the concepts with the most general intents and thus the largest extents. At the head of the lattice we can see the concepts with the most specific intents and thus the smallest extents.

There is a strong relationship between  FCA  and closed item-sets mining that aims at finding a set of nonredundant hypotheses investigated in the work of [24]. This is because a formal concept in  FCA  can be seen as a closed item-set in their formalism. The work of [25] also confirmed this relationship and explained that a concept in  FCA  expresses a maximal set of examples that shares all elements of a maximal set of features (rules) and vice versa. We will get back to the maximality property when discussing the relationship between  FCA  and trees at the end of the section.

### 4.2. Representing Rules with Multiple Concept Lattices

Assuming that we have a fixed set of first-order rules (or propositional rules) in the intents  *R*, we could extend the conventional  FCA  to two-class problem by simply extending the extent set  *X*  to  *X* ^+^, the set of examples belonging to the first class, and  *X* ^−^, the set of examples belonging the the second class. However, if each class is known to have a separate intent, as well as a separate extent the problem can be reformulated as follows.

Consider a set of first-order rules  *R* ^+^  and examples  *X* ^+^  of class  *λ* ^+^ ∈ Λ. Consider a set of first-order rules  *R* ^−^  and examples  *X* ^−^  of class  *λ* ^−^ ∈ Λ and a relation  *G*⊆*X* × *R*  such that  *X* = *X* ^+^ ∪ *X* ^−^, *R* = *R* ^+^ ∪ *R* ^−^. We say that  (*x* ^*s*^, *r* ^*s*^) ∈ *G* ^*s*^  if and only if example  *x* ^*s*^ ∈ *X* ^*s*^  is covered by rule  *r* ^*s*^ ∈ *R* ^*s*^, where  *s* ∈ {+, −}.  A formal context  *K*  is then the triple  (*X*, *R*, *G*). The formal concept and concept lattice are defined similarly as in Section 4.1.

The above can be seen as merging two formal contexts  *K* ^+^ = (*X* ^+^, *R* ^+^, *G* ^+^)  with  *K* ^−^ = (*X* ^−^, *R* ^−^, *G* ^−^)  to form the single formal context  *K*. Notice that we could find a case where  *x* ^+^  may be covered by  *r* ^−^  ; for  example, (*x* ^+^, *r* ^−^) ∉ *G* ^−^  or  (*x* ^+^, *r* ^−^) ∉ *G* ^+^. This may present a noise in the extents or an underfitting (overgenerality) in the intents. Another form of noise might occur if the same example  *x* ^+^  is also covered by  *r* ^+^,  for  example, (*x* ^+^, *r* ^+^) ∈ *G* ^+^, which suggests a conflict in the original theories induced for the positive and negative classes.

To this extent we explained how  FCA  can be used in a two-class scenario but it is not hard to see how such a formalism can be generalised for a multiclass scenario (having multiple theories for multiple classes) by introducing further formal contexts.

### 4.3. Representing Rules with a Single Concept Lattice

Having multiple formal contexts and their corresponding concept lattices may seem appropriate especially to visualise multiple one-versus-rest theories in ILP but apart from the high complexity of building multiple lattices, we are more interested to combine the models, visualise them, and study their collective performance. Therefore, a better solution would be to build a single concept lattice by taking into account all rules and examples as if the rules were generated from a single model learned over a multiclass problem. Consequently, the intent  *R*  corresponds to the set of rules induced for all the classes and the extent  *X*  corresponds to the set of all examples belonging to all the classes. Different colours can be used to distinguish the rules predicting different classes and similarly to distinguish examples belonging to various classes.

**Figure 6:**
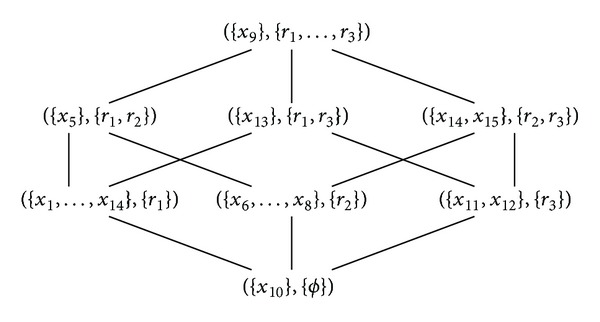
Concept lattice that corresponds to Example 5 and Table 1.

Figure 6 illustrates drawing a single formal concept lattice for Example 5 discussed above.

### 4.4. FCA and Rule Trees

Reference [26] amongst some others investigated inducing decision trees as selected paths from large concept lattices in a propositional domain. They regard the concept lattice as a collection of overlapping trees and the task is to search for the most accurate one in a classification context. In our case we employed  FCA  as a postlearning phase and we have a limited number of rules to represent. As a matter of fact, the formal concepts correspond to the leaves of a complete binary or rule tree as it is the case in  MRSI  method as can be seen in Theorem 9 and illustrated by Figure 6 and Figure 4. This is because the maximality property is maintained in the leaves of a  MRSI  tree. Nevertheless, this is not the case when it comes to  MRL  or  MRSU  because the maximality property is broken.

**Theorem 9**

MRSI's leaves are equivalent to formal concepts.

**Proof**

Let  *A*⊆*X*,  *B*⊆*R*, *B*′ = ⋂_*r*∈*B*_coverage(*r*)∖⋃_*r*∉*B*_coverage(*r*), and  *A*′ = ⋂_*x*∈*A*_  covered  By(*x*); then  *A*′ = *B*  (selected set of rules on nodes of a  MRSI  tree) and  *B*′ = *A*  (intersection set of examples covered by the selected set of rules that represents a  MRSI's leaf) satisfying the requirements of a formal concept  (*A*, *B*), where  coveredBy(*x*)  is a function that returns all rules covering an example  *x*  and  *A*  is the set of examples found in a leaf of the tree described by the set of all rules in  *B*  that apply to all examples in  *A*.

**Figure 7:**
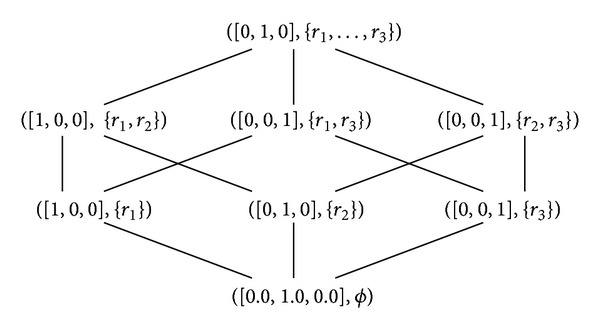
Probabilistic concept lattice that corresponds to Table 1 in Example 5.

Since we established the link between  MRSI's rule trees and concept lattices, it is possible to turn the concept lattice into a probabilistic classifier similarly to  MRSI  method by associating each internal node in the lattice by a probability distribution instead of the actual coverage promoting a probabilistic concept lattice as can be seen in Figure 7. The figure is useful in showing the probability distribution when one or multiple rules fire for a given example. Of course having the complete description of rules can be more useful but this is just an illustrative example and we have a limited space for drawing the concept lattice. Therefore, both  MRSI  and the probabilistic concept lattice can be used in the same way.

At this stage it is not obvious how we could take advantage of the formal concept lattice to be used with  MRL  and  MRSU  methods but the good news is that  MRSI  experimentally outperforms the other two methods when it comes to multiclass domains in terms of predictive accuracy and AUC (AUC is an abbreviation for the area under the ROC curve and used as a measure of predictive performance; for more details about AUC and ROC please see [27]) as will be shown in the next section.

## 5. Empirical Evaluation

In this section we evaluate and compare our proposed single multiclass theory learning methods (MRL,  MRSU, and  MRSI) over 6 multiclass data sets and 5 binary data sets (Table 2). We use Aleph as our base-learner, learning rules for each class in turn. We then turn the rules learned by Aleph into coherent multiclass models using the techniques proposed in Section 3. We compare the performance of our methods and  CN2  rule set method described above.

For each data set, cross-validated accuracies (Table 3) and AUCs (Table 4) were recorded. The  MRL  method does not produce class probabilities and hence produces a single point in a ROC plot; in this case, AUC boils down to the (unweighed) average of true positive and true negative rates.  MRSU,  MRSI, and  CN2  produce class probabilities and hence AUC evaluates their ranking performance in the usual way. A multiclass AUC is obtained by averaging each one-versus-rest AUC weighted by the class prior.

**Figure 8:**
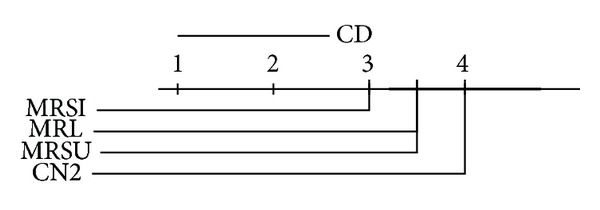
Posthoc test comparing the average rank of AUCs of  MRL,  MRSU,  MRSI, and  CN2  methods over the multiclass relational data sets.

**Figure 9:**
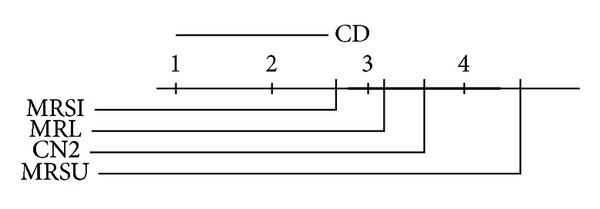
Posthoc test comparing the average rank of accuracies of  MRL,  MRSU,  MRSI, and  CN2  methods over the multiclass relational data sets.

**Figure 10:**
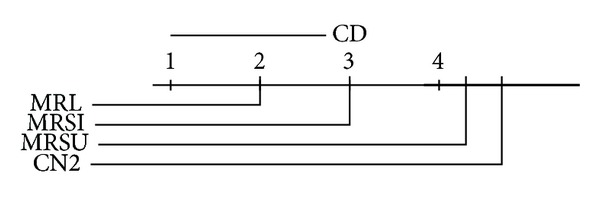
Posthoc test comparing the average rank of AUCs of  MRL,  MRSU,  MRSI, and  CN2  methods over the two-class relational data sets.

**Figure 11:**
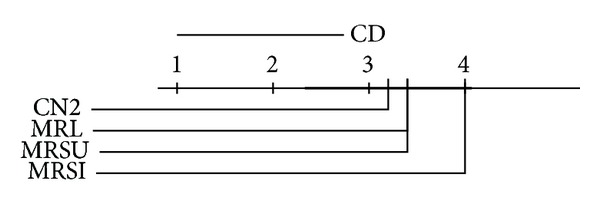
Posthoc test (no significance reported) comparing the average rank of accuracies of  MRL,  MRSU,  MRSI, and  CN2  methods over the two-class relational data sets.

We report the ranks (1 is best, 4 is worst) of the accuracies and AUCs on each data set. We use the Friedman significance test on these ranks at  *P* = 0.10  with Bonferroni-Dunn posthoc test on our three proposed methods. In the Friedman test we record wins and losses in the form of ranks and ignore the magnitude of these wins and losses. Graphical illustrations of the posthoc test results in the AUC and accuracy ranks are given in Figures 8, 9, 10, and 11 for the multiclass and the two-class data sets. The critical difference (CD) value is shown at the top of the figure. If the difference between two methods exceeds this value, then the methods are significantly different; otherwise, they are not. In the later case a thick black line will connect them together to indicate the insignificance difference between them. Note that the lower the rank is, the higher the performance is. By looking at the average performance rank and calculating the posthoc test and critical difference  (CD = 1.79), on the multiclass data sets,  MRSI  is significantly better than  CN2  on both accuracy and AUC, while  MRSU  performs significantly worse on AUC. If we take a look at the binary data sets (CD = 1.95), we can see that both  MRL  and  MRSI  are significantly outperforming  CN2  with respect to AUC, while no statistical significance is reported regarding their accuracies. The conclusion seems warranted that  MRSI  is preferable for multiclass data sets, while  MRL  is preferable for binary data sets.

For reference we also show the (multimodel) accuracy reported by Aleph, although this does not correspond to a coherent multiclass model and overemphasises the default rules. Also reported is the average positive recall, but this does not take proper account of rule overlaps.

## 6. Concluding Remarks

In this work we investigated the lack of reliability and consistency of the one-versus-rest technique on multiclass domains. We showed that we could build a simple and single multiclass model by combining the rules of all one-versus-rest models and turning them into a coherent multiclass classifier and we proposed three methods for that: Multiclass Rule List (MRL), Multiclass Rule Set Union (MRSU), and Multiclass Rule Set Intersection (MRSI).

Moreover we showed that we can adapt a graphical model with the help of Formal Concept Analysis (FCA) such that it can be used to explore the relationships and partial order between rules with respect to their coverage over the examples.

In Section 3 we illustrated our proposed multiclass methods in term of rule lists and rule trees and in Section 4 a connection between the  MRSI  method and a formal concept lattice was drawn. We pointed out that it is possible to use a formal concept lattice as a probabilistic classifier similarly to  MRSI  but with a simpler and more compact representation.

We showed that our proposed methods generate consistent and reliable multiclass predictions and experimentally produce significant results, with respect to accuracy and AUC, on both multiclass and binary domains when compared to the  CN2  method. When classification is made based on rule intersection,  MRSI, the best accuracies and AUCs were achieved taking the multiclass data sets into account. The rule list method seems to be suitable for two-class problems. The origin of this difference is the subject of ongoing investigations. The difference suggests that  MRL  benefits from having trees with larger leaves (i.e., Figure 3) to best decide one of two classes in two-class scenarios while this becomes a bit harder when having multiclass scenarios where  MRSI  method, reflecting trees with smaller leaves (i.e., Figure 4), tends to perform better.

## Figures and Tables

**Table 1:** Coverage of three rules over a problem of 15 examples *x*_1_,…, *x*_15_ belonging to three classes *λ*_1_,…, *λ*_3_.

Rule	Class predicted	Coverage	*χ* ^2^ score
*r*_1_	*λ*_1_	{*x*_1_,…, *x*_5_, *x*_9_, *x*_13_}	8.57
*r*_2_	*λ*_3_	{*x*_5_,…, *x*_9_, *x*_14_, *x*_15_}	3.75
*r*_3_	*λ*_3_	{*x*_9_, *x*_11_,…, *x*_15_}	11.67
Uncovered	*λ*_2_ (Majority of uncovered)	{*x*_10_}	N.A.

**Table 2:** Data sets used in the experiments. The group to the left is multi-class data sets while the group to the right is binary data sets. Starred data sets are propositional; the rest are relational.

Data set	Name	Class dist.
1	Car*	1210, 384, 69, 65
2	Diterpene	447, 355, 352, 155, 71
3	Ecoli*	143, 77, 52
4	English	50, 50, 50
5	Protein	116, 115, 77, 73
6	Scale*	288, 288, 49
7	Mutagenesis	125, 63
8	Amine (Alzheimer)	1026, 343
9	Choline (Alzheimer)	1026, 343
10	Scopolamine (Alzheimer)	1026, 343
11	Toxic (Alzheimer)	1026, 343

**Table 3:** Accuracies of our new multi-class methods (MRL, MRSU, and MRSI) compared to CN2 accuracy, with average ranks in brackets. The 6th column shows the multi-model accuracy as reported by Aleph, which is particularly optimistic for multi-class problems due to overemphasising the default rules. The rightmost column shows the average positive recall, which ignores the default rules but is still not equal to multi-class accuracy as conflicting predictions are not taken into account.

	MRL	MRSU	MRSI	CN2	Aleph standard	
	Multi-class accuracy				Multi-model accuracy	Average recall
Data set 1	81.43 (2.00)	81.32 (4.00)	**83.98 (1.00) **	81.38 (3.00)	86.90	82.18
Data set 2	83.70 (2.00)	83.55 (3.50)	**84.86 (1.00) **	83.55 (3.50)	91.52	82.91
Data set 3	**90.43 (1.00) **	86.77 (4.00)	89.75 (2.00)	88.92 (3.00)	90.27	86.46
Data set 4	60.67 (3.00)	58.00 (4.00)	**64.00 (1.00) **	62.67 (2.00)	72.44	48.00
Data set 5	80.48 (3.00)	80.69 (2.00)	79.70 (4.00)	**80.94 (1.00) **	89.91	70.82
Data set 6	80.64 (2.00)	72.51 (4.00)	**83.68 (1.00) **	76.20 (3.00)	79.04	71.20

Average	79.56 (2.17)	77.14 (3.58)	**80.99 (1.67) **	78.94 (2.58)	85.01	73.59

Data set 7	**77.06 (2.00) **	** 77.06 (2.00) **	76.55 (4.00)	**77.06 (2.00) **	73.97	73.97
Data set 8	60.18 (4.00)	60.91 (3.00)	**65.38 (1.00) **	60.98 (2.00)	77.06	77.06
Data set 9	**78.24 (1.00) **	76.07 (3.00)	77.14 (2.00)	75.55 (4.00)	60.11	60.18
Data set 10	**76.56 (2.00) **	** 76.56 (2.00) **	76.48 (4.00)	** 76.56 (2.00) **	76.56	76.56
Data set 11	74.95 (3.00)	75.02 (2.00)	74.59 (4.00)	**75.09 (1.00) **	74.80	74.80

Average	73.40 (2.40)	73.12 (2.40)	74.03 (3.00)	**73.05 (2.20) **	72.50	72.51

**Table 4:** Average one-versus-rest AUCs of our multi-class methods (MRL, MRSU, and MRSI) compared to CN2, with average ranks in brackets. The AUCs reported for Aleph are for reference only, as these arise from overemphasising the default rules.

	MRL	MRSU	MRSI	CN2	Aleph standard
Data set 1	**83.03 (1.00) **	75.2 (3.00)	73.92 (4.00)	75.39 (2.00)	82.80
Data set 2	88.72 (4.00)	**89.65 (1.00) **	88.90 (3.00)	89.58 (2.00)	88.66
Data set 3	91.97 (3.00)	92.63 (2.00)	**93.38 (1.00) **	91.43 (4.00)	86.78
Data set 4	70.50 (4.00)	72.67 (2.00)	**74.15 (1.00) **	72.60 (3.00)	66.33
Data set 5	87.28 (2.00)	86.62 (4.00)	**89.03 (1.00) **	86.63 (3.00)	83.29
Data set 6	**82.05 (1.00) **	74.03 (3.00)	81.41 (2.00)	73.27 (4.00)	76.38

Average	83.92 (2.50)	81.80 (2.50)	**83.46 (2.00) **	81.48 (3.00)	80.71

Data set 7	**64.03 (1.00) **	57.19 (4.00)	57.28 (2.00)	57.19 (3.00)	63.93
Data set 8	**60.77 (1.00) **	51.70 (3.00)	57.10 (2.00)	51.39 (4.00)	64.03
Data set 9	**74.48 (1.00) **	63.91 (3.00)	72.38 (2.00)	60.93 (4.00)	60.90
Data set 10	**55.07 (1.00) **	52.70 (3.50)	52.70 (2.00)	52.70 (3.50)	55.07
Data set 11	**65.46 (1.00) **	56.15 (3.00)	57.06 (2.00)	55.53 (4.00)	64.71

Average	**63.96 (1.00) **	56.33 (3.30)	59.30 (2.00)	55.55 (3.70)	61.73

## References

[1] Lavrač N, Sašo S, Žeroski D (1994). *Inductive Logic Programming: Techniques and Applications*.

[2] Nilsson NJ (1996). *Introduction To Machine Learning*.

[3] Quinlan JR, Cameron-Jones RM FOIL: a midterm report.

[4] Muggleton S Inverse entailment and progol.

[5] Srinivasan A (2001). The aleph manual.

[6] Mitchell T (1997). *Machine Learning*.

[7] Blockeel H, De Raedt L (1998). Top-down induction of first-order logical decision trees. *Artificial Intelligence*.

[8] De Raedt L, Van Laer W (1995). Inductive constraint logic. *Algorithmic Learning Theory*.

[9] Clark P, Niblett T (1989). The CN2 induction algorithm. *Machine Learning*.

[10] Dietterich TG, Bakiri G (1995). Solving multiclass learning problems via error-correcting output codes. *Artificial Intelligence Research*.

[11] Friedman JH (1996). Another approach to polychotomous classification.

[12] Hsu C-W, Lin C-J (2002). A comparison of methods for multiclass support vector machines. *Neural Networks*.

[13] Kijsirikul B, Ussivakul N, Meknavin S Adaptive directed acyclic graphs for multiclass classification.

[14] Platt JC, Cristianini N (2000). Large margin DAGs for multiclass classification. *Advances in Neural Information Processing Systems*.

[15] Rifkin R, Klautau A (2004). Defense of one-Vs-All classification. *Machine Learning Research*.

[16] Zadrozny B, Elkan C Transforming classifier scores into accurate multiclass probability estimates.

[17] Abudawood T, Flach P (2009). Evaluation measures for multi-class subgroup discovery. *European Conference on Machine Learning and Principles and Practice of Knowledge Discovery in Databases*.

[18] Abudawood T, Flach P Exploiting the high predictive power of multi-class subgroups.

[19] Abudawood T (2011). *Multi-Class Subgroup Discovery: Heuristics, Algorithms and Predictiveness [Ph.D. thesis]*.

[20] Belohlavek R, De Baets B, Outrata J, Vychodil V, Torra V, Narukawa Y, Yoshida Y (2007). Trees in concept lattices. *Modeling Decisions For Artificial Intelligence*.

[21] Kuznetsov SO (2004). Machine learning and formal concept analysis. *Concept Lattices*.

[22] Missaoui R, Nourine L, Renaud Y (2008). Generating positive and negative exact rules using formal concept analysis: problems and solutions. *Formal Concept Analysis*.

[23] Poelmans J, Elzinga P, Viaene S, Dedene G (2010). Formal concept analysis in knowledge discovery: a survey. *Lecture Notes in Computer Science*.

[24] Garriga GC, Kralj P, Lavrač N (2008). Closed sets for labeled data. *Journal of Machine Learning Research*.

[25] Pensa RG, Boulicaut J-F, Bandini S, Manzoni S Towards fault-tolerant formal concept analysis.

[26] Belohlavek R, De Baets B, Outrata J, Vychodil V (2009). Inducing decision trees via concept lattices. *International Journal of General Systems*.

[27] Fawcett T (2006). An introduction to ROC analysis. *Pattern Recognition Letters*.

